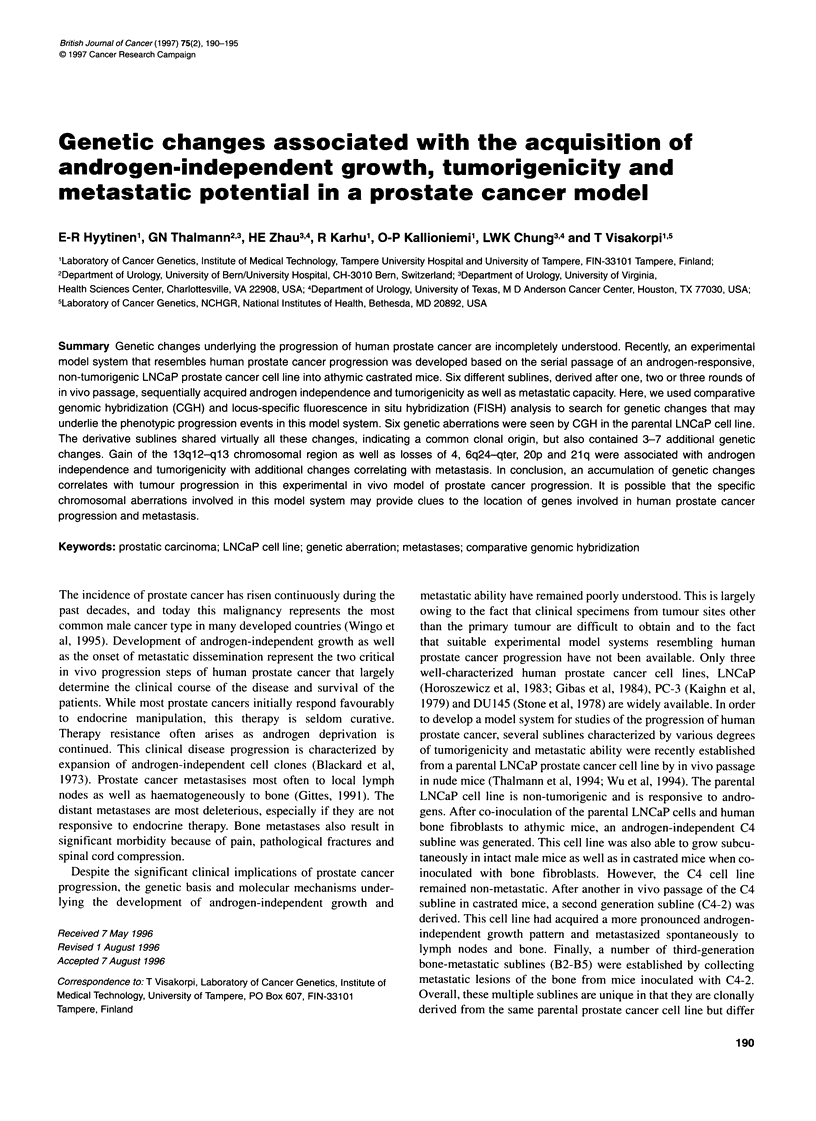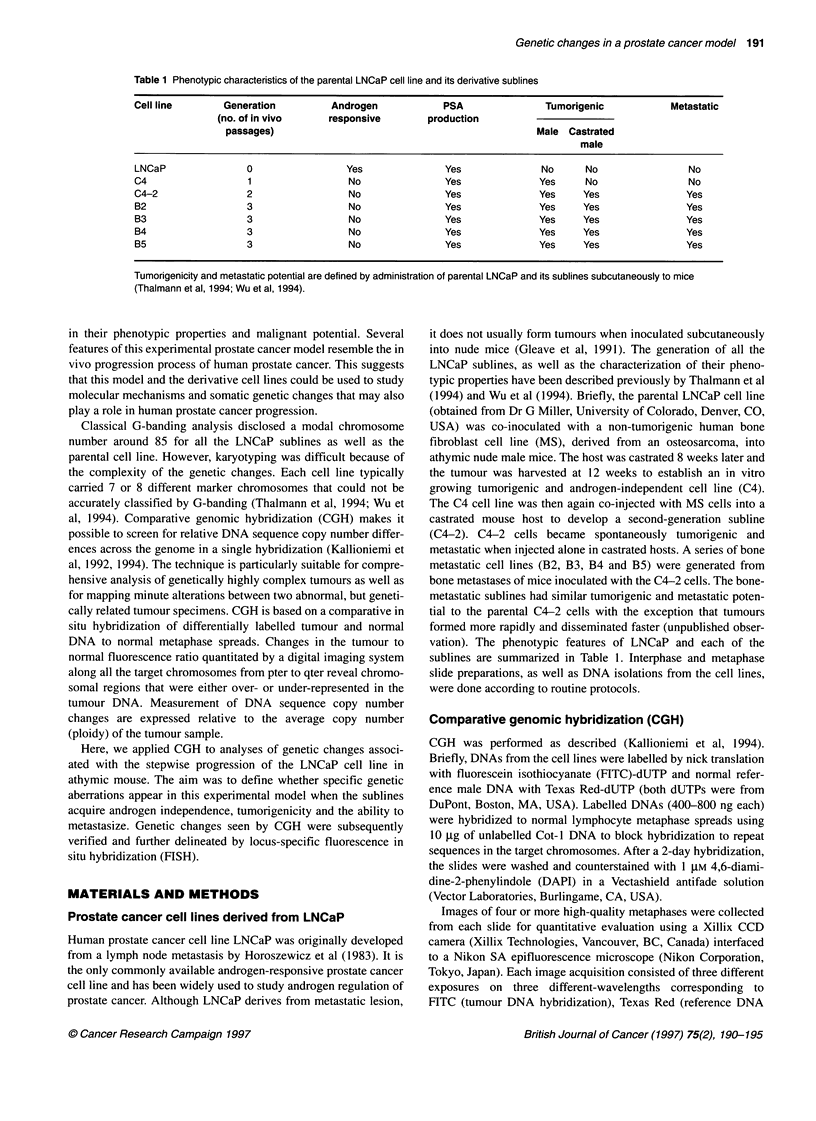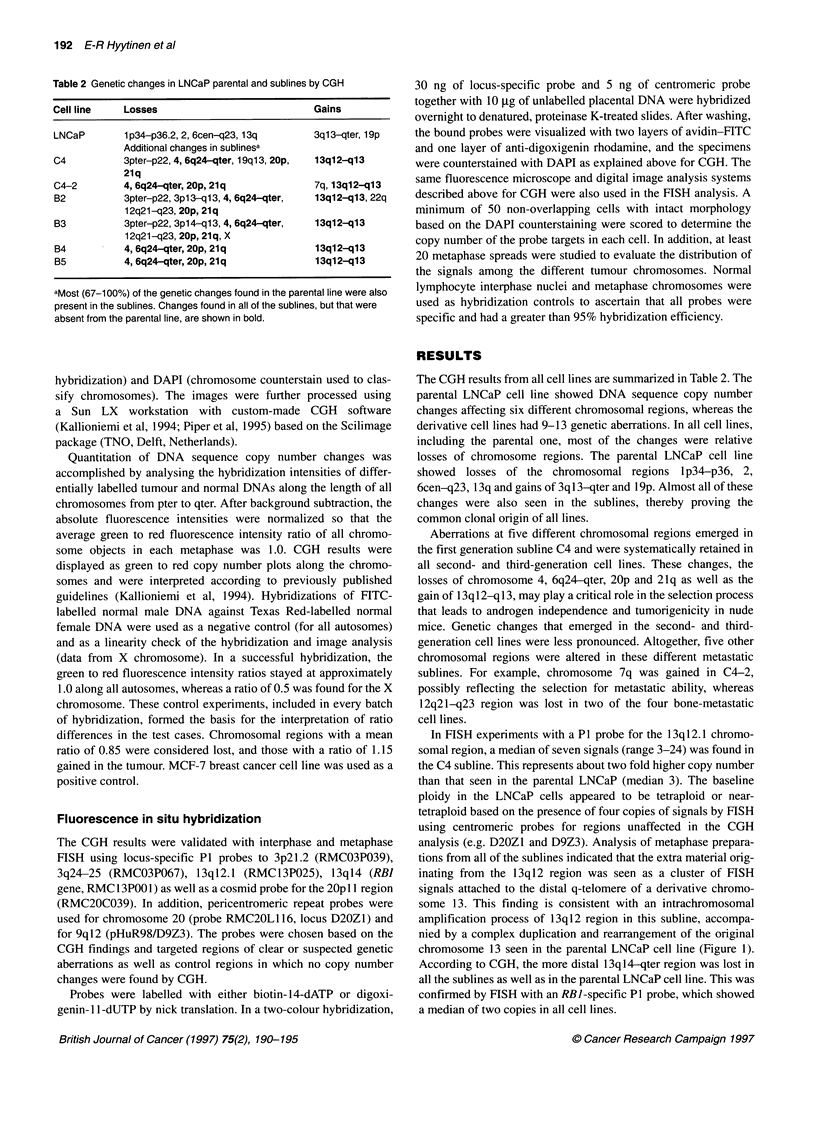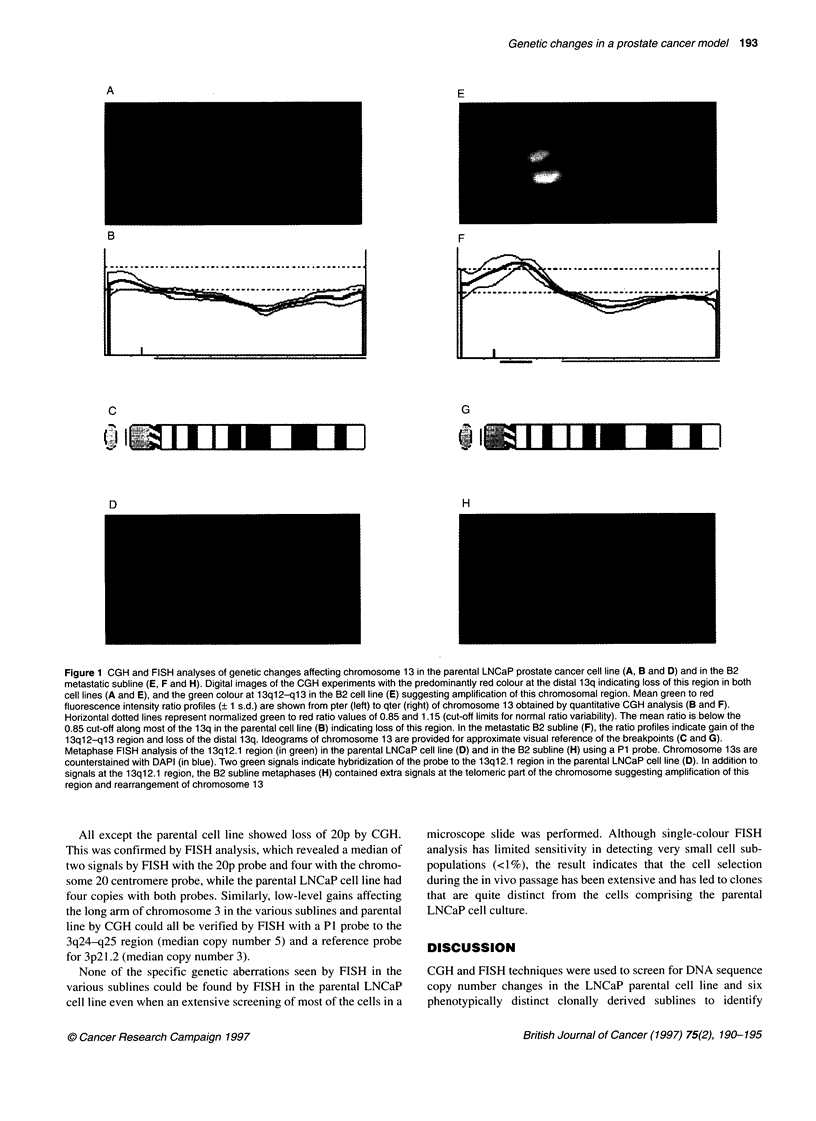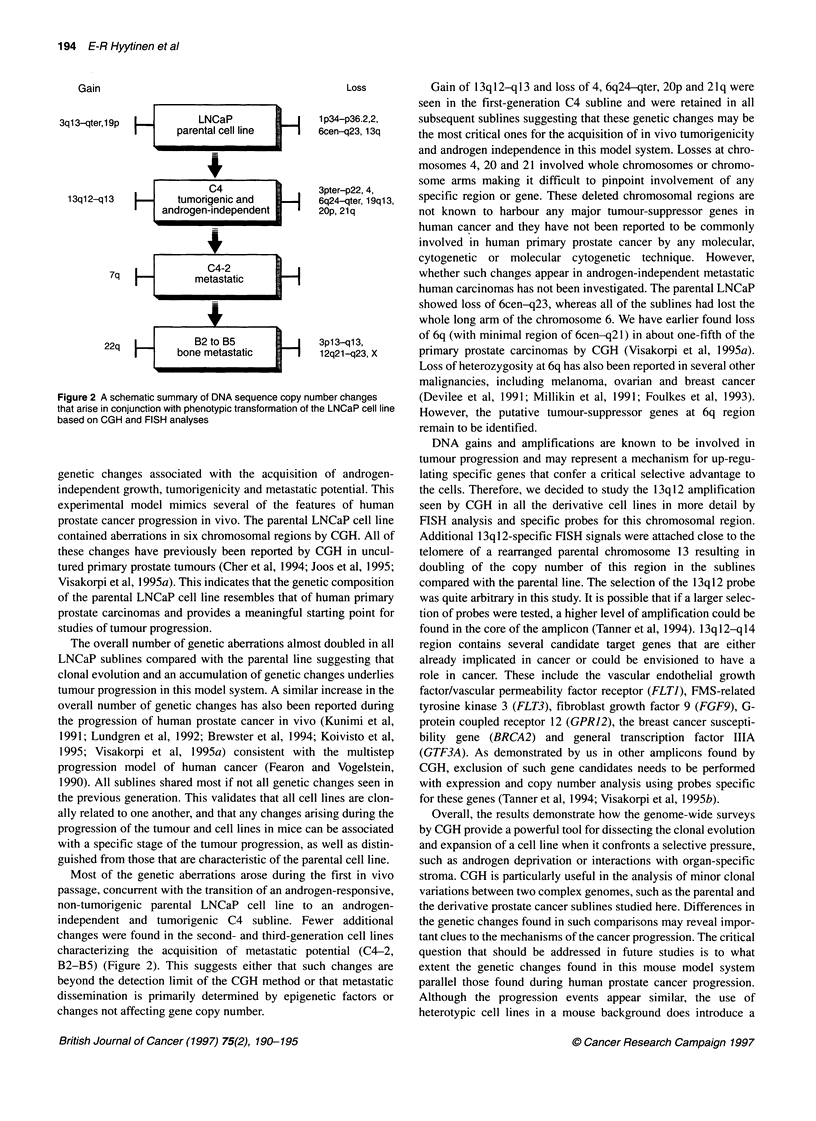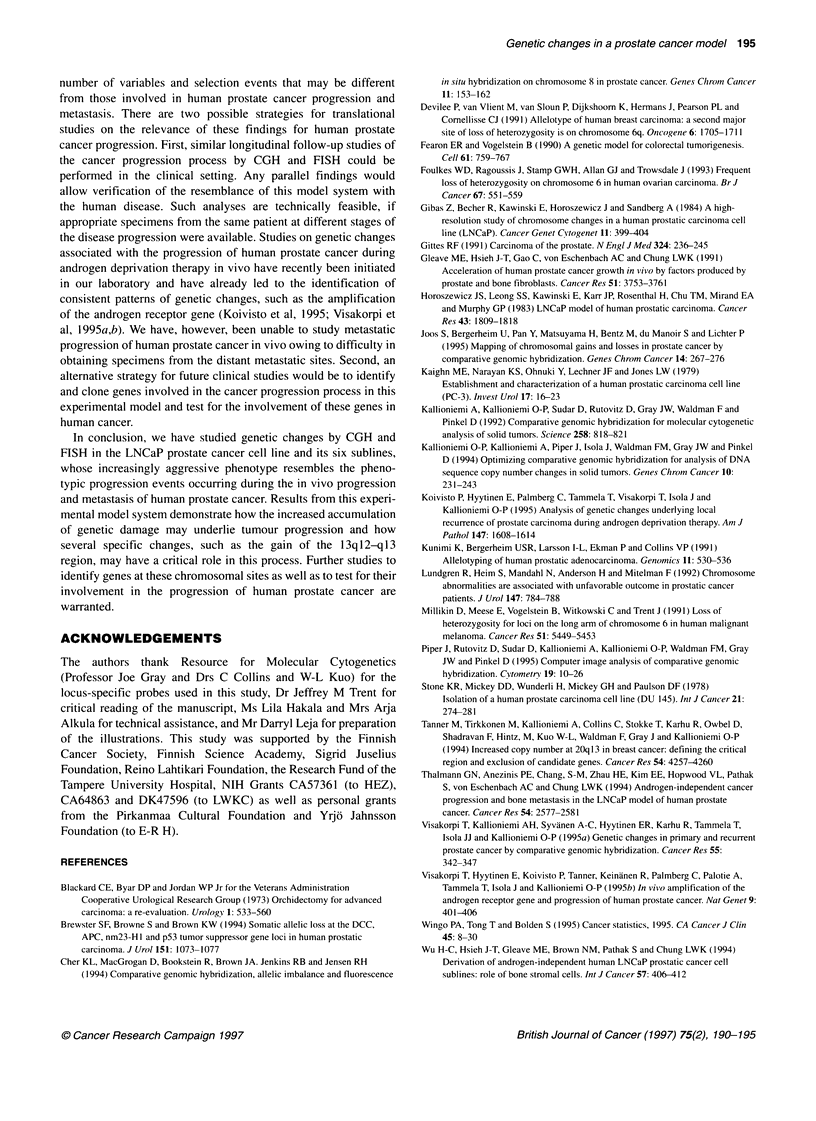# Genetic changes associated with the acquisition of androgen-independent growth, tumorigenicity and metastatic potential in a prostate cancer model.

**DOI:** 10.1038/bjc.1997.32

**Published:** 1997

**Authors:** E. R. Hyytinen, G. N. Thalmann, H. E. Zhau, R. Karhu, O. P. Kallioniemi, L. W. Chung, T. Visakorpi

**Affiliations:** Laboratory of Cancer Genetics, Tampere University Hospital, Finland.

## Abstract

**Images:**


British Journal of Cancer (1997) 75(2), 190–195
© 1997 Cancer Research Campaign

### Genetic changes associated with the acquisition of androgen-independent growth, tumorigenicity and metastatic potential in a prostate cancer model

E-R Hyytinen[1], GN Thalmann[2,3], HE Zhau[3,4], R Karhu[1], O-P Kallioniemi[1], LWK Chung[3,4] and T Visakorpi[1,5]

[1]Laboratory of Cancer Genetics, Institute of Medical Technology, Tampere University Hospital and University of Tampere, FIN-33101 Tampere, Finland; [2]Department of Urology, University of Bern/University Hospital, CH-3010 Bern, Switzerland; [3]Department of Urology, University of Virginia, Health Sciences Center, Charlottesville, VA 22908, USA; [4]Department of Urology, University of Texas, M D Anderson Cancer Center, Houston, TX 77030, USA; [5]Laboratory of Cancer Genetics, NCHGR, National Institutes of Health, Bethesda, MD 20892, USA

**Summary** Genetic changes underlying the progression of human prostate cancer are incompletely understood. Recently, an experimental model system that resembles human prostate cancer progression was developed based on the serial passage of an androgen-responsive, non-tumorigenic LNCaP prostate cancer cell line into athymic castrated mice. Six different sublines, derived after one, two or three rounds of in vivo passage, sequentially acquired androgen independence and tumorigenicity as well as metastatic capacity. Here, we used comparative genomic hybridization (CGH) and locus-specific fluorescence in situ hybridization (FISH) analysis to search for genetic changes that may underlie the phenotypic progression events in this model system. Six genetic aberrations were seen by CGH in the parental LNCaP cell line. The derivative sublines shared virtually all these changes, indicating a common clonal origin, but also contained 3–7 additional genetic changes. Gain of the 13q12–q13 chromosomal region as well as losses of 4, 6q24–qter, 20p and 21q were associated with androgen independence and tumorigenicity with additional changes correlating with metastasis. In conclusion, an accumulation of genetic changes correlates with tumour progression in this experimental in vivo model of prostate cancer progression. It is possible that the specific chromosomal aberrations involved in this model system may provide clues to the location of genes involved in human prostate cancer progression and metastasis.

**Keywords:** prostatic carcinoma; LNCaP cell line; genetic aberration; metastases; comparative genomic hybridization

The incidence of prostate cancer has risen continuously during the past decades, and today this malignancy represents the most common male cancer type in many developed countries (Wingo et al, 1995). Development of androgen-independent growth as well as the onset of metastatic dissemination represent the two critical in vivo progression steps of human prostate cancer that largely determine the clinical course of the disease and survival of the patients. While most prostate cancers initially respond favourably to endocrine manipulation, this therapy is seldom curative. Therapy resistance often arises as androgen deprivation is continued. This clinical disease progression is characterized by expansion of androgen-independent cell clones (Blackard et al, 1973). Prostate cancer metastasises most often to local lymph nodes as well as haematogeneously to bone (Gittes, 1991). The distant metastases are most deleterious, especially if they are not responsive to endocrine therapy. Bone metastases also result in significant morbidity because of pain, pathological fractures and spinal cord compression.

Despite the significant clinical implications of prostate cancer progression, the genetic basis and molecular mechanisms underlying the development of androgen-independent growth and metastatic ability have remained poorly understood. This is largely owing to the fact that clinical specimens from tumour sites other than the primary tumour are difficult to obtain and to the fact that suitable experimental model systems resembling human prostate cancer progression have not been available. Only three well-characterized human prostate cancer cell lines, LNCaP (Horoszewicz et al, 1983; Gibas et al, 1984), PC-3 (Kaighn et al, 1979) and DU145 (Stone et al, 1978) are widely available. In order to develop a model system for studies of the progression of human prostate cancer, several sublines characterized by various degrees of tumorigenicity and metastatic ability were recently established from a parental LNCaP prostate cancer cell line by in vivo passage in nude mice (Thalmann et al, 1994; Wu et al, 1994). The parental LNCaP cell line is non-tumorigenic and is responsive to androgens. After co-inoculation of the parental LNCaP cells and human bone fibroblasts to athymic mice, an androgen-independent C4 subline was generated. This cell line was also able to grow subcutaneously in intact male mice as well as in castrated mice when co-inoculated with bone fibroblasts. However, the C4 cell line remained non-metastatic. After another in vivo passage of the C4 subline in castrated mice, a second generation subline (C4-2) was derived. This cell line had acquired a more pronounced androgen-independent growth pattern and metastasized spontaneously to lymph nodes and bone. Finally, a number of third-generation bone-metastatic sublines (B2-B5) were established by collecting metastatic lesions of the bone from mice inoculated with C4-2. Overall, these multiple sublines are unique in that they are clonally derived from the same parental prostate cancer cell line but differ

*Received 7 May 1996*
*Revised 1 August 1996*
*Accepted 7 August 1996*

*Correspondence to:* T Visakorpi, Laboratory of Cancer Genetics, Institute of Medical Technology, University of Tampere, PO Box 607, FIN-33101 Tampere, Finland

Table 1 Phenotypic characteristics of the parental LNCaP cell line and its derivative sublines

| Cell line | Generation (no. of in vivo passages) | Androgen responsive | PSA production | Tumorigenic | | Metastatic |
|---|---|---|---|---|---|---|
| | | | | Male | Castrated male | |
| LNCaP | 0 | Yes | Yes | No | No | No |
| C4 | 1 | No | Yes | Yes | No | No |
| C4–2 | 2 | No | Yes | Yes | Yes | Yes |
| B2 | 3 | No | Yes | Yes | Yes | Yes |
| B3 | 3 | No | Yes | Yes | Yes | Yes |
| B4 | 3 | No | Yes | Yes | Yes | Yes |
| B5 | 3 | No | Yes | Yes | Yes | Yes |

Tumorigenicity and metastatic potential are defined by administration of parental LNCaP and its sublines subcutaneously to mice (Thalmann et al, 1994; Wu et al, 1994).

in their phenotypic properties and malignant potential. Several features of this experimental prostate cancer model resemble the in vivo progression process of human prostate cancer. This suggests that this model and the derivative cell lines could be used to study molecular mechanisms and somatic genetic changes that may also play a role in human prostate cancer progression.

Classical G-banding analysis disclosed a modal chromosome number around 85 for all the LNCaP sublines as well as the parental cell line. However, karyotyping was difficult because of the complexity of the genetic changes. Each cell line typically carried 7 or 8 different marker chromosomes that could not be accurately classified by G-banding (Thalmann et al, 1994; Wu et al, 1994). Comparative genomic hybridization (CGH) makes it possible to screen for relative DNA sequence copy number differences across the genome in a single hybridization (Kallioniemi et al, 1992, 1994). The technique is particularly suitable for comprehensive analysis of genetically highly complex tumours as well as for mapping minute alterations between two abnormal, but genetically related tumour specimens. CGH is based on a comparative in situ hybridization of differentially labelled tumour and normal DNA to normal metaphase spreads. Changes in the tumour to normal fluorescence ratio quantitated by a digital imaging system along all the target chromosomes from pter to qter reveal chromosomal regions that were either over- or under-represented in the tumour DNA. Measurement of DNA sequence copy number changes are expressed relative to the average copy number (ploidy) of the tumour sample.

Here, we applied CGH to analyses of genetic changes associated with the stepwise progression of the LNCaP cell line in athymic mouse. The aim was to define whether specific genetic aberrations appear in this experimental model when the sublines acquire androgen independence, tumorigenicity and the ability to metastasize. Genetic changes seen by CGH were subsequently verified and further delineated by locus-specific fluorescence in situ hybridization (FISH).

### MATERIALS AND METHODS

### Prostate cancer cell lines derived from LNCaP

Human prostate cancer cell line LNCaP was originally developed from a lymph node metastasis by Horoszewicz et al (1983). It is the only commonly available androgen-responsive prostate cancer cell line and has been widely used to study androgen regulation of prostate cancer. Although LNCaP derives from metastatic lesion, it does not usually form tumours when inoculated subcutaneously into nude mice (Gleave et al, 1991). The generation of all the LNCaP sublines, as well as the characterization of their phenotypic properties have been described previously by Thalmann et al (1994) and Wu et al (1994). Briefly, the parental LNCaP cell line (obtained from Dr G Miller, University of Colorado, Denver, CO, USA) was co-inoculated with a non-tumorigenic human bone fibroblast cell line (MS), derived from an osteosarcoma, into athymic nude male mice. The host was castrated 8 weeks later and the tumour was harvested at 12 weeks to establish an in vitro growing tumorigenic and androgen-independent cell line (C4). The C4 cell line was then again co-injected with MS cells into a castrated mouse host to develop a second-generation subline (C4–2). C4–2 cells became spontaneously tumorigenic and metastatic when injected alone in castrated hosts. A series of bone metastatic cell lines (B2, B3, B4 and B5) were generated from bone metastases of mice inoculated with the C4–2 cells. The bone-metastatic sublines had similar tumorigenic and metastatic potential to the parental C4–2 cells with the exception that tumours formed more rapidly and disseminated faster (unpublished observation). The phenotypic features of LNCaP and each of the sublines are summarized in Table 1. Interphase and metaphase slide preparations, as well as DNA isolations from the cell lines, were done according to routine protocols.

### Comparative genomic hybridization (CGH)

CGH was performed as described (Kallioniemi et al, 1994). Briefly, DNAs from the cell lines were labelled by nick translation with fluorescein isothiocyanate (FITC)-dUTP and normal reference male DNA with Texas Red-dUTP (both dUTPs were from DuPont, Boston, MA, USA). Labelled DNAs (400–800 ng each) were hybridized to normal lymphocyte metaphase spreads using 10 μg of unlabelled Cot-1 DNA to block hybridization to repeat sequences in the target chromosomes. After a 2-day hybridization, the slides were washed and counterstained with 1 μM 4,6-diamidine-2-phenylindole (DAPI) in a Vectashield antifade solution (Vector Laboratories, Burlingame, CA, USA).

Images of four or more high-quality metaphases were collected from each slide for quantitative evaluation using a Xillix CCD camera (Xillix Technologies, Vancouver, BC, Canada) interfaced to a Nikon SA epifluorescence microscope (Nikon Corporation, Tokyo, Japan). Each image acquisition consisted of three different exposures on three different-wavelengths corresponding to FITC (tumour DNA hybridization), Texas Red (reference DNA

© Cancer Research Campaign 1997

Table 2 Genetic changes in LNCaP parental and sublines by CGH

| Cell line | Losses | Gains |
|---|---|---|
| LNCaP | 1p34–p36.2, 2, 6cen–q23, 13q | 3q13–qter, 19p |
| | Additional changes in sublines[a] | |
| C4 | 3pter–p22, **4, 6q24–qter**, 19q13, **20p, 21q** | **13q12–q13** |
| C4–2 | **4, 6q24–qter, 20p, 21q** | 7q, **13q12–q13** |
| B2 | 3pter–p22, 3p13–q13, **4, 6q24–qter**, 12q21–q23, **20p, 21q** | **13q12–q13**, 22q |
| B3 | 3pter–p22, 3p14–q13, **4, 6q24–qter**, 12q21–q23, **20p, 21q**, X | **13q12–q13** |
| B4 | **4, 6q24–qter, 20p, 21q** | **13q12–q13** |
| B5 | **4, 6q24–qter, 20p, 21q** | **13q12–q13** |

[a]Most (67–100%) of the genetic changes found in the parental line were also present in the sublines. Changes found in all of the sublines, but that were absent from the parental line, are shown in bold.

hybridization) and DAPI (chromosome counterstain used to classify chromosomes). The images were further processed using a Sun LX workstation with custom-made CGH software (Kallioniemi et al, 1994; Piper et al, 1995) based on the Scilimage package (TNO, Delft, Netherlands).

Quantitation of DNA sequence copy number changes was accomplished by analysing the hybridization intensities of differentially labelled tumour and normal DNAs along the length of all chromosomes from pter to qter. After background subtraction, the absolute fluorescence intensities were normalized so that the average green to red fluorescence intensity ratio of all chromosome objects in each metaphase was 1.0. CGH results were displayed as green to red copy number plots along the chromosomes and were interpreted according to previously published guidelines (Kallioniemi et al, 1994). Hybridizations of FITC-labelled normal male DNA against Texas Red-labelled normal female DNA were used as a negative control (for all autosomes) and as a linearity check of the hybridization and image analysis (data from X chromosome). In a successful hybridization, the green to red fluorescence intensity ratios stayed at approximately 1.0 along all autosomes, whereas a ratio of 0.5 was found for the X chromosome. These control experiments, included in every batch of hybridization, formed the basis for the interpretation of ratio differences in the test cases. Chromosomal regions with a mean ratio of 0.85 were considered lost, and those with a ratio of 1.15 gained in the tumour. MCF-7 breast cancer cell line was used as a positive control.

### Fluorescence in situ hybridization

The CGH results were validated with interphase and metaphase FISH using locus-specific P1 probes to 3p21.2 (RMC03P039), 3q24–25 (RMC03P067), 13q12.1 (RMC13P025), 13q14 (*RB1* gene, RMC13P001) as well as a cosmid probe for the 20p11 region (RMC20C039). In addition, pericentromeric repeat probes were used for chromosome 20 (probe RMC20L116, locus D20Z1) and for 9q12 (pHuR98/D9Z3). The probes were chosen based on the CGH findings and targeted regions of clear or suspected genetic aberrations as well as control regions in which no copy number changes were found by CGH.

Probes were labelled with either biotin-14-dATP or digoxigenin-11-dUTP by nick translation. In a two-colour hybridization, 30 ng of locus-specific probe and 5 ng of centromeric probe together with 10 μg of unlabelled placental DNA were hybridized overnight to denatured, proteinase K-treated slides. After washing, the bound probes were visualized with two layers of avidin–FITC and one layer of anti-digoxigenin rhodamine, and the specimens were counterstained with DAPI as explained above for CGH. The same fluorescence microscope and digital image analysis systems described above for CGH were also used in the FISH analysis. A minimum of 50 non-overlapping cells with intact morphology based on the DAPI counterstaining were scored to determine the copy number of the probe targets in each cell. In addition, at least 20 metaphase spreads were studied to evaluate the distribution of the signals among the different tumour chromosomes. Normal lymphocyte interphase nuclei and metaphase chromosomes were used as hybridization controls to ascertain that all probes were specific and had a greater than 95% hybridization efficiency.

### RESULTS

The CGH results from all cell lines are summarized in Table 2. The parental LNCaP cell line showed DNA sequence copy number changes affecting six different chromosomal regions, whereas the derivative cell lines had 9–13 genetic aberrations. In all cell lines, including the parental one, most of the changes were relative losses of chromosome regions. The parental LNCaP cell line showed losses of the chromosomal regions 1p34–p36, 2, 6cen–q23, 13q and gains of 3q13–qter and 19p. Almost all of these changes were also seen in the sublines, thereby proving the common clonal origin of all lines.

Aberrations at five different chromosomal regions emerged in the first generation subline C4 and were systematically retained in all second- and third-generation cell lines. These changes, the losses of chromosome 4, 6q24–qter, 20p and 21q as well as the gain of 13q12–q13, may play a critical role in the selection process that leads to androgen independence and tumorigenicity in nude mice. Genetic changes that emerged in the second- and third-generation cell lines were less pronounced. Altogether, five other chromosomal regions were altered in these different metastatic sublines. For example, chromosome 7q was gained in C4–2, possibly reflecting the selection for metastatic ability, whereas 12q21–q23 region was lost in two of the four bone-metastatic cell lines.

In FISH experiments with a P1 probe for the 13q12.1 chromosomal region, a median of seven signals (range 3–24) was found in the C4 subline. This represents about two fold higher copy number than that seen in the parental LNCaP (median 3). The baseline ploidy in the LNCaP cells appeared to be tetraploid or near-tetraploid based on the presence of four copies of signals by FISH using centromeric probes for regions unaffected in the CGH analysis (e.g. D20Z1 and D9Z3). Analysis of metaphase preparations from all of the sublines indicated that the extra material originating from the 13q12 region was seen as a cluster of FISH signals attached to the distal q-telomere of a derivative chromosome 13. This finding is consistent with an intrachromosomal amplification process of 13q12 region in this subline, accompanied by a complex duplication and rearrangement of the original chromosome 13 seen in the parental LNCaP cell line (Figure 1). According to CGH, the more distal 13q14–qter region was lost in all the sublines as well as in the parental LNCaP cell line. This was confirmed by FISH with an *RB1*-specific P1 probe, which showed a median of two copies in all cell lines.

© Cancer Research Campaign 1997

**Figure 1** CGH and FISH analyses of genetic changes affecting chromosome 13 in the parental LNCaP prostate cancer cell line (**A**, **B** and **D**) and in the B2 metastatic subline (**E**, **F** and **H**). Digital images of the CGH experiments with the predominantly red colour at the distal 13q indicating loss of this region in both cell lines (**A** and **E**), and the green colour at 13q12–q13 in the B2 cell line (**E**) suggesting amplification of this chromosomal region. Mean green to red fluorescence intensity ratio profiles (± 1 s.d.) are shown from pter (left) to qter (right) of chromosome 13 obtained by quantitative CGH analysis (**B** and **F**). Horizontal dotted lines represent normalized green to red ratio values of 0.85 and 1.15 (cut-off limits for normal ratio variability). The mean ratio is below the 0.85 cut-off along most of the 13q in the parental cell line (**B**) indicating loss of this region. In the metastatic B2 subline (**F**), the ratio profiles indicate gain of the 13q12–q13 region and loss of the distal 13q. Ideograms of chromosome 13 are provided for approximate visual reference of the breakpoints (**C** and **G**). Metaphase FISH analysis of the 13q12.1 region (in green) in the parental LNCaP cell line (**D**) and in the B2 subline (**H**) using a P1 probe. Chromosome 13s are counterstained with DAPI (in blue). Two green signals indicate hybridization of the probe to the 13q12.1 region in the parental LNCaP cell line (**D**). In addition to signals at the 13q12.1 region, the B2 subline metaphases (**H**) contained extra signals at the telomeric part of the chromosome suggesting amplification of this region and rearrangement of chromosome 13

All except the parental cell line showed loss of 20p by CGH. This was confirmed by FISH analysis, which revealed a median of two signals by FISH with the 20p probe and four with the chromosome 20 centromere probe, while the parental LNCaP cell line had four copies with both probes. Similarly, low-level gains affecting the long arm of chromosome 3 in the various sublines and parental line by CGH could all be verified by FISH with a P1 probe to the 3q24–q25 region (median copy number 5) and a reference probe for 3p21.2 (median copy number 3).

None of the specific genetic aberrations seen by FISH in the various sublines could be found by FISH in the parental LNCaP cell line even when an extensive screening of most of the cells in a microscope slide was performed. Although single-colour FISH analysis has limited sensitivity in detecting very small cell subpopulations (<1%), the result indicates that the cell selection during the in vivo passage has been extensive and has led to clones that are quite distinct from the cells comprising the parental LNCaP cell culture.

### DISCUSSION

CGH and FISH techniques were used to screen for DNA sequence copy number changes in the LNCaP parental cell line and six phenotypically distinct clonally derived sublines to identify

© Cancer Research Campaign 1997

**Figure 2** A schematic summary of DNA sequence copy number changes that arise in conjunction with phenotypic transformation of the LNCaP cell line based on CGH and FISH analyses genetic changes associated with the acquisition of androgen-independent growth, tumorigenicity and metastatic potential. This experimental model mimics several of the features of human prostate cancer progression in vivo. The parental LNCaP cell line contained aberrations in six chromosomal regions by CGH. All of these changes have previously been reported by CGH in uncultured primary prostate tumours (Cher et al, 1994; Joos et al, 1995; Visakorpi et al, 1995*a*). This indicates that the genetic composition of the parental LNCaP cell line resembles that of human primary prostate carcinomas and provides a meaningful starting point for studies of tumour progression.

The overall number of genetic aberrations almost doubled in all LNCaP sublines compared with the parental line suggesting that clonal evolution and an accumulation of genetic changes underlies tumour progression in this model system. A similar increase in the overall number of genetic changes has also been reported during the progression of human prostate cancer in vivo (Kunimi et al, 1991; Lundgren et al, 1992; Brewster et al, 1994; Koivisto et al, 1995; Visakorpi et al, 1995*a*) consistent with the multistep progression model of human cancer (Fearon and Vogelstein, 1990). All sublines shared most if not all genetic changes seen in the previous generation. This validates that all cell lines are clonally related to one another, and that any changes arising during the progression of the tumour and cell lines in mice can be associated with a specific stage of the tumour progression, as well as distinguished from those that are characteristic of the parental cell line.

Most of the genetic aberrations arose during the first in vivo passage, concurrent with the transition of an androgen-responsive, non-tumorigenic parental LNCaP cell line to an androgen-independent and tumorigenic C4 subline. Fewer additional changes were found in the second- and third-generation cell lines characterizing the acquisition of metastatic potential (C4–2, B2–B5) (Figure 2). This suggests either that such changes are beyond the detection limit of the CGH method or that metastatic dissemination is primarily determined by epigenetic factors or changes not affecting gene copy number.

Gain of 13q12–q13 and loss of 4, 6q24–qter, 20p and 21q were seen in the first-generation C4 subline and were retained in all subsequent sublines suggesting that these genetic changes may be the most critical ones for the acquisition of in vivo tumorigenicity and androgen independence in this model system. Losses at chromosomes 4, 20 and 21 involved whole chromosomes or chromosome arms making it difficult to pinpoint involvement of any specific region or gene. These deleted chromosomal regions are not known to harbour any major tumour-suppressor genes in human cancer and they have not been reported to be commonly involved in human primary prostate cancer by any molecular, cytogenetic or molecular cytogenetic technique. However, whether such changes appear in androgen-independent metastatic human carcinomas has not been investigated. The parental LNCaP showed loss of 6cen–q23, whereas all of the sublines had lost the whole long arm of the chromosome 6. We have earlier found loss of 6q (with minimal region of 6cen–q21) in about one-fifth of the primary prostate carcinomas by CGH (Visakorpi et al, 1995*a*). Loss of heterozygosity at 6q has also been reported in several other malignancies, including melanoma, ovarian and breast cancer (Devilee et al, 1991; Millikin et al, 1991; Foulkes et al, 1993). However, the putative tumour-suppressor genes at 6q region remain to be identified.

DNA gains and amplifications are known to be involved in tumour progression and may represent a mechanism for up-regulating specific genes that confer a critical selective advantage to the cells. Therefore, we decided to study the 13q12 amplification seen by CGH in all the derivative cell lines in more detail by FISH analysis and specific probes for this chromosomal region. Additional 13q12-specific FISH signals were attached close to the telomere of a rearranged parental chromosome 13 resulting in doubling of the copy number of this region in the sublines compared with the parental line. The selection of the 13q12 probe was quite arbitrary in this study. It is possible that if a larger selection of probes were tested, a higher level of amplification could be found in the core of the amplicon (Tanner et al, 1994). 13q12–q14 region contains several candidate target genes that are either already implicated in cancer or could be envisioned to have a role in cancer. These include the vascular endothelial growth factor/vascular permeability factor receptor (*FLT1*), FMS-related tyrosine kinase 3 (*FLT3*), fibroblast growth factor 9 (*FGF9*), G-protein coupled receptor 12 (*GPR12*), the breast cancer susceptibility gene (*BRCA2*) and general transcription factor IIIA (*GTF3A*). As demonstrated by us in other amplicons found by CGH, exclusion of such gene candidates needs to be performed with expression and copy number analysis using probes specific for these genes (Tanner et al, 1994; Visakorpi et al, 1995*b*).

Overall, the results demonstrate how the genome-wide surveys by CGH provide a powerful tool for dissecting the clonal evolution and expansion of a cell line when it confronts a selective pressure, such as androgen deprivation or interactions with organ-specific stroma. CGH is particularly useful in the analysis of minor clonal variations between two complex genomes, such as the parental and the derivative prostate cancer sublines studied here. Differences in the genetic changes found in such comparisons may reveal important clues to the mechanisms of the cancer progression. The critical question that should be addressed in future studies is to what extent the genetic changes found in this mouse model system parallel those found during human prostate cancer progression. Although the progression events appear similar, the use of heterotypic cell lines in a mouse background does introduce a

© Cancer Research Campaign 1997

number of variables and selection events that may be different from those involved in human prostate cancer progression and metastasis. There are two possible strategies for translational studies on the relevance of these findings for human prostate cancer progression. First, similar longitudinal follow-up studies of the cancer progression process by CGH and FISH could be performed in the clinical setting. Any parallel findings would allow verification of the resemblance of this model system with the human disease. Such analyses are technically feasible, if appropriate specimens from the same patient at different stages of the disease progression were available. Studies on genetic changes associated with the progression of human prostate cancer during androgen deprivation therapy in vivo have recently been initiated in our laboratory and have already led to the identification of consistent patterns of genetic changes, such as the amplification of the androgen receptor gene (Koivisto et al, 1995; Visakorpi et al, 1995*a*,*b*). We have, however, been unable to study metastatic progression of human prostate cancer in vivo owing to difficulty in obtaining specimens from the distant metastatic sites. Second, an alternative strategy for future clinical studies would be to identify and clone genes involved in the cancer progression process in this experimental model and test for the involvement of these genes in human cancer.

In conclusion, we have studied genetic changes by CGH and FISH in the LNCaP prostate cancer cell line and its six sublines, whose increasingly aggressive phenotype resembles the phenotypic progression events occurring during the in vivo progression and metastasis of human prostate cancer. Results from this experimental model system demonstrate how the increased accumulation of genetic damage may underlie tumour progression and how several specific changes, such as the gain of the 13q12–q13 region, may have a critical role in this process. Further studies to identify genes at these chromosomal sites as well as to test for their involvement in the progression of human prostate cancer are warranted.

### ACKNOWLEDGEMENTS

The authors thank Resource for Molecular Cytogenetics (Professor Joe Gray and Drs C Collins and W-L Kuo) for the locus-specific probes used in this study, Dr Jeffrey M Trent for critical reading of the manuscript, Ms Lila Hakala and Mrs Arja Alkula for technical assistance, and Mr Darryl Leja for preparation of the illustrations. This study was supported by the Finnish Cancer Society, Finnish Science Academy, Sigrid Juselius Foundation, Reino Lahtikari Foundation, the Research Fund of the Tampere University Hospital, NIH Grants CA57361 (to HEZ), CA64863 and DK47596 (to LWKC) as well as personal grants from the Pirkanmaa Cultural Foundation and Yrjö Jahnsson Foundation (to E-R H).

### REFERENCES

Blackard CE, Byar DP and Jordan WP Jr for the Veterans Administration Cooperative Urological Research Group (1973) Orchidectomy for advanced carcinoma: a re-evaluation. *Urology* **1**: 533–560

Brewster SF, Browne S and Brown KW (1994) Somatic allelic loss at the DCC, APC, nm23-H1 and p53 tumor suppressor gene loci in human prostatic carcinoma. *J Urol* **151**: 1073–1077

Cher KL, MacGrogan D, Bookstein R, Brown JA. Jenkins RB and Jensen RH (1994) Comparative genomic hybridization, allelic imbalance and fluorescence *in situ* hybridization on chromosome 8 in prostate cancer. *Genes Chrom Cancer* **11**: 153–162

Devilee P, van Vlient M, van Sloun P, Dijkshoorn K, Hermans J, Pearson PL and Cornellisse CJ (1991) Allelotype of human breast carcinoma: a second major site of loss of heterozygosity is on chromosome 6q. *Oncogene* **6**: 1705–1711

Fearon ER and Vogelstein B (1990) A genetic model for colorectal tumorigenesis. *Cell* **61**: 759–767

Foulkes WD, Ragoussis J, Stamp GWH, Allan GJ and Trowsdale J (1993) Frequent loss of heterozygosity on chromosome 6 in human ovarian carcinoma. *Br J Cancer* **67**: 551–559

Gibas Z, Becher R, Kawinski E, Horoszewicz J and Sandberg A (1984) A high-resolution study of chromosome changes in a human prostatic carcinoma cell line (LNCaP). *Cancer Genet Cytogenet* **11**: 399–404

Gittes RF (1991) Carcinoma of the prostate. *N Engl J Med* **324**: 236–245

Gleave ME, Hsieh J-T, Gao C, von Eschenbach AC and Chung LWK (1991) Acceleration of human prostate cancer growth *in vivo* by factors produced by prostate and bone fibroblasts. *Cancer Res* **51**: 3753–3761

Horoszewicz JS, Leong SS, Kawinski E, Karr JP, Rosenthal H, Chu TM, Mirand EA and Murphy GP (1983) LNCaP model of human prostatic carcinoma. *Cancer Res* **43**: 1809–1818

Joos S, Bergerheim U, Pan Y, Matsuyama H, Bentz M, du Manoir S and Lichter P (1995) Mapping of chromosomal gains and losses in prostate cancer by comparative genomic hybridization. *Genes Chrom Cancer* **14**: 267–276

Kaighn ME, Narayan KS, Ohnuki Y, Lechner JF and Jones LW (1979) Establishment and characterization of a human prostatic carcinoma cell line (PC-3). *Invest Urol* **17**: 16–23

Kallioniemi A, Kallioniemi O-P, Sudar D, Rutovitz D, Gray JW, Waldman F and Pinkel D (1992) Comparative genomic hybridization for molecular cytogenetic analysis of solid tumors. *Science* **258**: 818–821

Kallioniemi O-P, Kallioniemi A, Piper J, Isola J, Waldman FM, Gray JW and Pinkel D (1994) Optimizing comparative genomic hybridization for analysis of DNA sequence copy number changes in solid tumors. *Genes Chrom Cancer* **10**: 231–243

Koivisto P, Hyytinen E, Palmberg C, Tammela T, Visakorpi T, Isola J and Kallioniemi O-P (1995) Analysis of genetic changes underlying local recurrence of prostate carcinoma during androgen deprivation therapy. *Am J Pathol* **147**: 1608–1614

Kunimi K, Bergerheim USR, Larsson I-L, Ekman P and Collins VP (1991) Allelotyping of human prostatic adenocarcinoma. *Genomics* **11**: 530–536

Lundgren R, Heim S, Mandahl N, Anderson H and Mitelman F (1992) Chromosome abnormalities are associated with unfavorable outcome in prostatic cancer patients. *J Urol* **147**: 784–788

Millikin D, Meese E, Vogelstein B, Witkowski C and Trent J (1991) Loss of heterozygosity for loci on the long arm of chromosome 6 in human malignant melanoma. *Cancer Res* **51**: 5449–5453

Piper J, Rutovitz D, Sudar D, Kallioniemi A, Kallioniemi O-P, Waldman FM, Gray JW and Pinkel D (1995) Computer image analysis of comparative genomic hybridization. *Cytometry* **19**: 10–26

Stone KR, Mickey DD, Wunderli H, Mickey GH and Paulson DF (1978) Isolation of a human prostate carcinoma cell line (DU 145). *Int J Cancer* **21**: 274–281

Tanner M, Tirkkonen M, Kallioniemi A, Collins C, Stokke T, Karhu R, Owbel D, Shadravan F, Hintz, M, Kuo W-L, Waldman F, Gray J and Kallioniemi O-P (1994) Increased copy number at 20q13 in breast cancer: defining the critical region and exclusion of candidate genes. *Cancer Res* **54**: 4257–4260

Thalmann GN, Anezinis PE, Chang, S-M, Zhau HE, Kim EE, Hopwood VL, Pathak S, von Eschenbach AC and Chung LWK (1994) Androgen-independent cancer progression and bone metastasis in the LNCaP model of human prostate cancer. *Cancer Res* **54**: 2577–2581

Visakorpi T, Kallioniemi AH, Syvänen A-C, Hyytinen ER, Karhu R, Tammela T, Isola JJ and Kallioniemi O-P (1995*a*) Genetic changes in primary and recurrent prostate cancer by comparative genomic hybridization. *Cancer Res* **55**: 342–347

Visakorpi T, Hyytinen E, Koivisto P, Tanner, Keinänen R, Palmberg C, Palotie A, Tammela T, Isola J and Kallioniemi O-P (1995*b*) *In vivo* amplification of the androgen receptor gene and progression of human prostate cancer. *Nat Genet* **9**: 401–406

Wingo PA, Tong T and Bolden S (1995) Cancer statistics, 1995. *CA Cancer J Clin* **45**: 8–30

Wu H-C, Hsieh J-T, Gleave ME, Brown NM, Pathak S and Chung LWK (1994) Derivation of androgen-independent human LNCaP prostatic cancer cell sublines: role of bone stromal cells. *Int J Cancer* **57**: 406–412

© Cancer Research Campaign 1997